# MicroRNAs in Podocyte Injury in Diabetic Nephropathy

**DOI:** 10.3389/fgene.2020.00993

**Published:** 2020-08-25

**Authors:** Hiroki Ishii, Shohei Kaneko, Katsunori Yanai, Akinori Aomatsu, Keiji Hirai, Susumu Ookawara, Kenichi Ishibashi, Yoshiyuki Morishita

**Affiliations:** ^1^Division of Nephrology, First Department of Integrated Medicine, Saitama Medical Center, Jichi Medical University, Saitama, Japan; ^2^Department of Medical Physiology, Meiji Pharmaceutical University, Kiyose, Japan

**Keywords:** podocyte injury, diabetic mellitus, microRNA, epigenetics, biomarker

## Abstract

Diabetic nephropathy is one of the major complications of diabetes mellitus and is the leading cause of end-stage renal disease worldwide. Podocyte injury contributes to the development of diabetic nephropathy. However, the molecules that regulate podocyte injury in diabetic nephropathy have not been fully clarified. MicroRNAs (miRNAs) are small non-coding RNAs that can inhibit the translation of target messenger RNAs. Previous reports have described alteration of the expression levels of many miRNAs in cultured podocyte cells stimulated with a high glucose concentration and podocytes in rodent models of diabetic nephropathy. The associations between podocyte injury and miRNA expression levels in blood, urine, and kidney in patients with diabetic nephropathy have also been reported. Moreover, modulation of the expression of several miRNAs has been shown to have protective effects against podocyte injury in diabetic nephropathy in cultured podocyte cells *in vitro* and in rodent models of diabetic nephropathy *in vivo*. Therefore, this review focuses on miRNAs in podocyte injury in diabetic nephropathy, with regard to their potential as biomarkers and miRNA modulation as a therapeutic option.

## Introduction

Diabetic nephropathy is one of the most severe microvascular complications, which affects over 40% of patients with diabetes mellitus (Fineberg et al., 2013). It is the leading cause of end-stage renal disease, which requires renal replacement therapy including dialysis and renal transplantation (Conserva et al., 2013). Diabetic nephropathy develops via multiple metabolic and hemodynamic pathways stimulated by persistent hyperglycemia. Various pathological changes such as abnormal mesangial expansion, glomerular hypertrophy, GBM thickening, and tubulointerstitial fibrosis are observed in diabetic nephropathy (Dronavalli et al., 2008). Additionally, podocyte injury has been shown to contribute to the development of diabetic nephropathy (Dalla Vestra et al., 2003; Susztak et al., 2006). Podocytes are highly differentiated glomerular epithelial cells attached to the outer surface of the GBM (Wolf et al., 2005). They play critical roles in maintaining the integrity of the glomerular filtration barrier (Wolf et al., 2005). Previous studies reported that, even at an early stage of diabetic nephropathy, the number of podocytes is drastically decreased, resulting in loss of the filtration barrier ability (Wolf et al., 2005; Fogo, 2011). Another study reported that podocyte injuries including the phenotypic change called podocyte mesenchymal transition, associated with disruption of podocyte architectural integrity and increased podocyte apoptosis, were observed in diabetic nephropathy (Shankland, 2006). However, the molecules that modulate these podocyte injuries in diabetic nephropathy have not been fully clarified.

MicroRNAs (miRNAs), which are single-stranded, short (19–25 bases), non-coding RNAs, have been drawing attention as essential regulators of gene expression by repressing the translation of or degrading target messenger RNAs (mRNA) by RNA interference (Ambros, 2004; Cheng et al., 2020). Substantial evidence has revealed the roles of miRNAs in the development and progression of various diseases including diabetic nephropathy (Lorenzen et al., 2011). Furthermore, alterations of miRNA levels serve not only as diagnostic biomarkers, but also as therapeutic targets in various diseases, such as cancer and certain inflammatory diseases (Mouradian, 2012; Trionfini et al., 2015; Hosseinahli et al., 2018). Previous studies have also shown that miRNAs play pivotal roles in the development and progression of diabetic nephropathy, indicating that they may represent potential biomarkers and therapeutic options for diabetic nephropathy (Dewanjee and Bhattacharjee, 2018; Gholaminejad et al., 2018). This review focuses on miRNAs and their potential as biomarkers and therapeutic options in podocyte injury in diabetic nephropathy.

## The Production Process of miRNA *In Vivo*

Single-stranded miRNA is initially transcribed from genomic DNA by RNA polymerase II in the nucleus (Lee et al., 2003). Then, this single-stranded RNA forms a hairpin structure called pri-miRNA by binding complementary sequences. Next, Drosha, an RNase III, cleaves the hairpin moiety of pri-miRNA. Subsequently, precursor miRNA (60–70 bases) is produced as an intermediate product (Denli et al., 2004). The produced precursor miRNA is mainly transported from the nucleus to the cytoplasm by exportin-5 (Okada et al., 2009). Then, the precursor miRNA is cleaved by the RNase III Dicer to short-form double-stranded RNA (21–24 bases) (Zhang et al., 2004). This double-stranded RNA is incorporated into the RNA-induced silencing complex (RISC) consisting of Ago proteins (Treiber et al., 2019). Then, double-stranded miRNA is dissolved into two single-stranded miRNAs (Treiber et al., 2019), after which the unstable single-stranded RNA is degraded. Finally, the residual stable single-stranded miRNA with RISC binds target messenger RNAs, resulting in inhibition of the translation of target mRNAs (Treiber et al., 2019).

Drosha or Dicer may cleave miRNA precursors at different points, as previously reported (Liang et al., 2017), after which miRNA variants called isomiRNAs are generated (Landgraf et al., 2007). IsomiRNAs are a few bases away from the 5′ or 3′ end of the reference sequences of miRNA, which have been registered in miRBase (Landgraf et al., 2007). The reference miRNA sequences are usually most highly expressed; however, certain isomiRNAs increase their expression level more than reference miRNAs under certain conditions. It has been suggested that at least some isomiRNAs may affect target selection, stability of miRNA, and loading ability (Guo and Chen, 2014).

## Mechanism of the Development of Podocyte Injury in Diabetic Nephropathy

**FIGURE 1:**
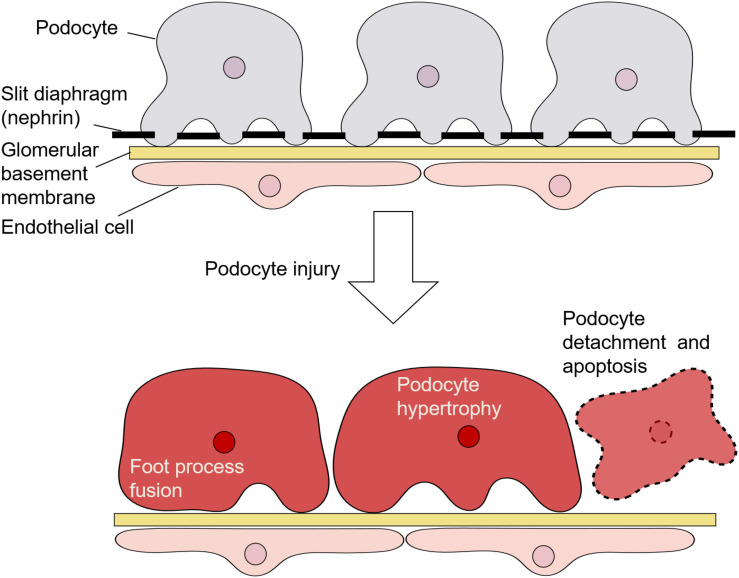
Architecture of podocytes and podocyte injury.

Podocytes are highly specialized epithelial cells located on the surface of the GBM; they have a complicated cellular architecture with a cell body and numerous foot processes (Figure 1) (Perico et al., 2016). Adjacent foot processes of podocytes are connected by an intercellular junction called the slit diaphragm, which serves as a barrier to regulate the passage of macromolecules from the blood (Wartiovaara et al., 2004). A podocyte protein called nephrin, which is a transmembrane receptor molecule, is a central component in the formation and maintenance of the slit diaphragm (Welsh and Saleem, 2010; Martin and Jones, 2018).

Morphologically, injury of podocytes is characterized by their hypertrophy and detachment from the GBM, as well as foot process fusion and/or effacement with the dysregulation of nephrin (Figure 1) (Perico et al., 2016). Such injury is responsible for proteinuria, the accumulation of extracellular matrix components, and glomerulosclerosis (Shankland, 2006). Podocyte loss is considered to be an irreversible event associated with a decline in glomerular filtration function (Perico et al., 2016). Additionally, podocyte depletion and structural changes are important features in the pathogenesis of diabetic nephropathy (Pichaiwong et al., 2013) and are suggested to be predictors of the progression of this disease (Lee et al., 2015; Puelles and Bertram, 2015).

Podocyte injury has been reported to be induced by multiple signaling pathways in diabetic nephropathy (Kawanami et al., 2016). For example, a previous study reported that TGF-β_1_ and Smad7 directly induce podocyte apoptosis in diabetic nephropathy (Schiffer et al., 2001). Unlike Smad7, TGF-β_1_ also induces podocyte apoptosis via the activation of p38 mitogen-activated protein (MAP) kinase and bcl-2-associated X protein (Bax), which activates the caspase-3 apoptotic pathway (Derynck and Zhang, 2003). TGF-β_1_ increases mitochondrial membrane potential and oxygen consumption rate, inducing increases in the generation of ROS and podocyte apoptosis (Abe et al., 2013).

Mammalian target of rapamycin (mTOR) is also an important factor for high glucose-induced podocyte injury. For example, recent studies have shown that mTOR complex1 (mTORC1) activity is increased in podocytes in diabetic nephropathy (Godel et al., 2011; Inoki et al., 2011). High glucose causes podocyte apoptosis through the stimulation of oxidative stress and ROS generation with the activation of mTORC1 (Kitada et al., 2017). Indeed, an mTORC1 inhibitor, rapamycin, was shown to suppress podocyte injury caused by high glucose in diabetic nephropathy (Li et al., 2019). In addition, genetic deletion of mTORC1 in mouse podocytes was found to induce proteinuria and progressive glomerulosclerosis (Godel et al., 2011). However, curtailing mTORC1 signaling in mice by genetically reducing the copy number of mTORC1 in podocytes was shown to ameliorate the progression of glomerular disease in diabetic nephropathy (Godel et al., 2011). These results suggested the need for tightly balanced mTOR activity in podocyte homeostasis in diabetic nephropathy (Godel et al., 2011; Li et al., 2019).

The Janus kinase/signal transducer and activator of transcription (JAK-STAT) signaling pathway has also been reported to play important roles in diabetic nephropathy (Marrero et al., 2006). In glomerular cells including podocytes, this pathway was shown to be activated in patients with even early-stage diabetic nephropathy (Berthier et al., 2009). OE of JAK2 in transgenic diabetic nephropathy model mice was shown to lead to a significant reduction in podocyte density (Zhang et al., 2017). In contrast, treatment with a JAK1/2 inhibitor, baricitinib, was shown to reduce albuminuria in patients with diabetic nephropathy (Zhang et al., 2017).

## Changes of miRNA Expression in Cultured Podocyte Cells Stimulated With a High Glucose Concentration, Rodent Model of Diabetic Nephropathy, and Patients With Diabetic Nephropathy

We searched for and reviewed miRNAs reported to be involved in podocyte injury in diabetic nephropathy, with regard to their potential as biomarkers and the possibility of modulating miRNAs as a therapeutic approach. Several studies have examined the changes of expression levels of miRNAs in cultured podocyte cells stimulated with a high glucose concentration *in vitro*, and in podocytes in rodent models with diabetic nephropathy *in vivo* (Table 1). The expression levels of 11 different miRNAs (miRNA-20b, -21, -27a, -29c, -134-5p, 193a, -195, -217, -218, -503, -770-5p) were shown to increase while the expression levels of 14 different miRNAs (miRNA-16-5p, -23b, -25, -29a, -30s, -34a, -34c,-93, -130a-3p, -145-5p, -146a, -301a-3p, -423-5p, -874) were shown to decrease in cultured podocytes *in vitro* or in podocytes in rodent models of diabetic nephropathy *in vivo* (Tables 1, 2) (Long et al., 2010, 2011; Chen et al., 2011, 2018; Lin et al., 2014; Liu et al., 2015, 2016, 2017; Badal et al., 2016; Yang et al., 2016; Zhang et al., 2016, 2019; Zhao et al., 2016; Guo et al., 2017; Kolling et al., 2017; Lee et al., 2017; Sun et al., 2017; Wang et al., 2017, 2018; Zhou et al., 2017; Jiang et al., 2018; Mishra et al., 2018; Qian et al., 2018; Xu et al., 2018; Yao et al., 2018; Duan et al., 2019; Zha et al., 2019; Wei et al., 2020). The associations between podocyte injury and miRNA expression levels in blood, urine, and kidney in patients with diabetic nephropathy were also reported (Table 2) (Long et al., 2011; Zhao et al., 2016; Guo et al., 2017; Kolling et al., 2017; Lee et al., 2017; Liu et al., 2017; Wang et al., 2017; Zhou et al., 2017; Xu et al., 2018; Ming et al., 2019). Furthermore, two different miRNAs (miRNA-21, -29c) in blood, one (miRNA-21) in urine and three (miRNA-21, -27a, -182-5p) in kidney tissues, were shown to increase, while two different miRNAs (miRNA-23b, -25) in blood, two (miRNA-23b, -29c) in urine, and three (miRNA-29c,-146a,-423-5p) in kidney tissues were shown to decrease in accordance with podocyte injuries in patients with diabetic nephropathy. These lines of evidence suggest that miRNAs are potential biomarkers of podocyte injury in diabetic nephropathy.

**TABLE 1:** Associations between podocyte injury and miRNA expression levels in cultured podocyte cells and rodent models of diabetic nephropathy.

miRNAs	Expression patterns	Sample	Predicted target mRNA	References
miRNA-16-5p	↓	Cultured human podocyte	VEGFA	Duan et al., 2019
miRNA-20b	↑	Conditionally immortalized cultured mouse podocytes	SIRT7	Wang et al., 2017
miRNA-21	↑	Conditionally immortalized cultured mouse podocytes. Kidney (KK-Ay mice received high-fat diets). Serum (KK-Ay mice received high-fat diets)	TIMP3 Cdc25a Cdk6	Kolling et al., 2017; Chen et al., 2018; Wang et al., 2018
miRNA-23b	↓	Cultured mouse podocytes. Kidney (STZ-induced diabetic nephropathy mice, db/db).	G3BP2	Zhao et al., 2016
miRNA-25	↓	Cultured mouse podocytes. Kidney (STZ-induced diabetic nephropathy mice, db/db). Serum (db/db).	Cdc42	Liu et al., 2017
miRNA-27a	↑	Conditionally immortalized cultured murine podocyte. Glomeruli (STZ-induced diabetic nephropathy rats). Plasma (STZ-induced diabetic nephropathy rats).	PPAR-γ	Zhou et al., 2017
miRNA-29a	↓	Primary cultured mouse podocytes. Immortalized cultured mouse podocyte. Glomeruli (STZ-induced diabetic nephropathy mice).	HDAC	Lin et al., 2014
miRNA-29c	↑	Conditionally immortalized cultured mouse podocytes. Glomeruli (db/db).	TTP Spry1	Long et al., 2011; Guo et al., 2017
miRNA-30s	↓	Conditionally immortalized cultured mouse podocytes. Glomeruli (STZ-induced diabetic nephropathy rats).	MTDH	Liu et al., 2016
miRNA-34a	↓	Conditionally immortalized cultured mouse podocytes.	Notch-1	Zhang et al., 2016
miRNA-34c	↓	Conditionally immortalized cultured mouse podocytes.	Notch1 Jagged1	Liu et al., 2015
miRNA-93	↓	Conditionally immortalized cultured mouse podocytes. Glomeruli (db/db).	VEGF-A Msk2	Long et al., 2010; Badal et al., 2016
miRNA-130a-3p	↓	Conditionally immortalized cultured mouse podocytes.	TNF-α	Jiang et al., 2018
miRNA-134-5p	↑	Conditionally immortalized cultured human podocyte. Kidney (db/db).	Bcl-2	Qian et al., 2018
miRNA-145-5p	↓	Cultured mouse podocytes.	Notch1	Wei et al., 2020
miRNA-146a	↓	Glomeruli.	ErbB4 Notch-1	Lee et al., 2017
miRNA-193a	↑	Conditionally immortalized cultured human podocytes.	APOL1	Mishra et al., 2018
miRNA-195	↑	Conditionally immortalized cultured mouse podocyte Glomeruli (db/db).	Bcl2	Chen et al., 2011
miRNA-217	↑	Cultured mouse podocytes.	PTEN	Sun et al., 2017
miRNA-218	↑	Conditionally immortalized cultured mouse podocytes.	HO-1	Yang et al., 2016
miRNA-301a-3p	↓	Conditionally immortalized cultured mouse podocytes.	TNF-α	Jiang et al., 2018
miRNA-423-5p	↓	Conditionally immortalized cultured mouse podocytes.	Nox4	Xu et al., 2018
miRNA-503	↑	Cultured mouse podocytes.	E2F3	Zha et al., 2019
miRNA-770-5p	↑	Cultured mouse podocyte.	TRIAP1	Zhang et al., 2019
miRNA-874	↓	Conditionally immortalized cultured mouse podocytes. Kidney (STZ-induced diabetic nephropathy rats).	TLR-4	Yao et al., 2018

**TABLE 2:** Associations between podocyte injury and miRNA expression levels in blood, urine, and kidney in patients with diabetic nephropathy.

miRNA	Expression pattern	Sample	Predicted target mRNA	References
miRNA-21	↑ ↑ ↑	Plasma Urine Kidney	TIMP3	Kolling et al., 2017
miRNA-23b	↓ ↓	Serum Urine	G3BP2	Zhao et al., 2016
miRNA-25	↓	Serum	CDC 42 Rap1a/b	Liu et al., 2017
miRNA-27a	↑	Kidney	PPAR-γ, activated β- catenin signaling	Zhou et al., 2017
miRNA-29c	↑ ↓ ↓	Plasma Urine Renal tissue	TTP Spry1	Long et al., 2011; Guo et al., 2017
miRNA-146a	↓	Kidney	ErbB4 Notch-1	Lee et al., 2017
miRNA-182-5p	↑	Kidney (podocyte)	CD2AP	Ming et al., 2019
miRNA-423-5p	↓	kidney	Nox4	Xu et al., 2018

## Methods of Modulation of miRNA Expression Levels on Podocyte in Diabetic Nephropathy

Several vectors such as liposome nanoparticles and viral vectors with miRNAs, as well as chemical modifications of miRNAs, are employed to modulate miRNA expression levels on podocytes in diabetic nephropathy. miRNA-874 with adenoviral vectors was shown to ameliorate podocyte injury by overexpressing miRNA in diabetic nephropathy mouse models *in vivo* (Yao et al., 2018). In addition, chemically modified double-stranded miRNAs of miRNA-23b and miRNA-25 were shown to inhibit podocyte injury in diabetic nephropathy mouse models *in vivo* (Zhao et al., 2016; Liu et al., 2017). However, there are still unresolved aspects of treatment of podocyte injury with miRNAs with/without vectors, such as efficient cellular uptake and intracellular re-release, as well as targeted delivery (Bartoszewski and Sikorski, 2019).

## The Effects of Each miRNA Expression Levels on Podocyte Injury in Diabetic Nephropathy

**FIGURE 2:**
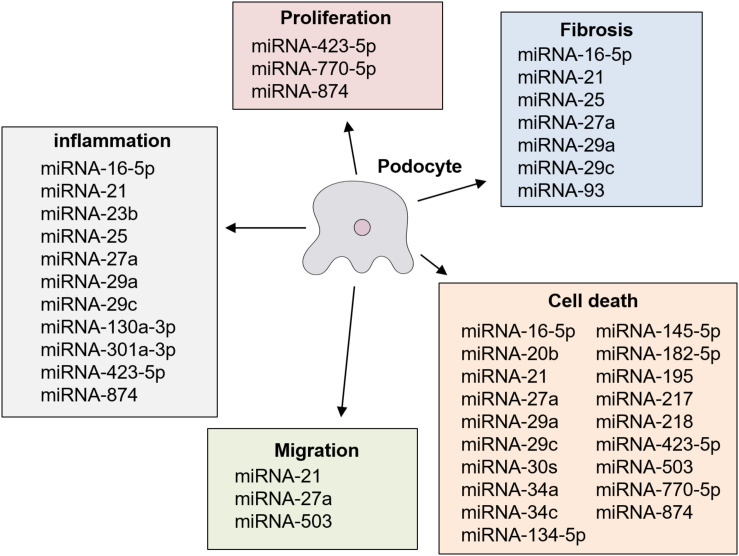
Roles of miRNA in podocyte injury in diabetic nephropathy.

Several previous studies reported that the OE or KD of different miRNAs had significant effects on podocyte injury in diabetic nephropathy in cultured podocyte cells stimulated with a high glucose concentration *in vitro*, and in rodent models with diabetic nephropathy *in vivo* (Tables 3A,B) (Long et al., 2010, 2011; Chen et al., 2011, 2018; Lin et al., 2014; Liu et al., 2015, 2016, 2017; Badal et al., 2016; Yang et al., 2016; Zhang et al., 2016, 2019; Zhao et al., 2016; Guo et al., 2017; Kolling et al., 2017; Lee et al., 2017; Sun et al., 2017; Wang et al., 2017, 2018; Zhou et al., 2017; Jiang et al., 2018; Mishra et al., 2018; Qian et al., 2018; Xu et al., 2018; Yao et al., 2018; Duan et al., 2019; Ming et al., 2019; Zha et al., 2019; Wei et al., 2020). These specific miRNAs are described below. The results of previous studies regarding modulating effects of miRNA on podocyte injury are summarized in Figure 2. miRNAs have been shown to be a novel class of key regulators in various biological processes in podocyte injury such as apoptosis, fibrosis, inflammation, migration, and proliferation (Figure 2).

**TABLE 3a:** Effects of changes of miRNA expression levels on podocyte injury in diabetic nephropathy *in vitro.*

miRNA	Model	Treatment	Biological effects	Target mRNA	References
miRNA-16-5p	Cultured human podocyte	OE	Caspase 3 ↓, Bax ↓, α-SMA ↓, nephrin ↑, TNF-α↓, TGF-β1 ↓, MCP-1 ↓,	VEGFA	Duan et al., 2019
miRNA-20b	Cultured mouse podocyte	KD	Apoptosis ↓, Caspase 3 ↓	SIRT7	Wang et al., 2017
miRNA-21	Cultured mouse podocyte	OE KD	Nephrin ↓, α-SMA ↑, TGF-β1 ↑, Smad3 ↑, Smad7 ↓, β-catenin ↑, Migration ↓	Wnt/β-catenin pathway and the TGF-β1/Smads pathway. PTEN	Kolling et al., 2017; Wang et al., 2018
miRNA-27a	Cultured mouse podocyte	KD	β-catenin ↓, Snail1 ↓, α-SMA ↓, Podocine ↑, Migration ↓, Invasion ↓, Apoptosis ↓	PPAR-γ	Zhou et al., 2017
miRNA-29a	Cultured mouse podocyte	OE	Ubiquitinated nephrin ↓, Nephrin ↑, Acetylated nephrin ↑, WT-1 ↑ Apoptosis ↓	HDAC4	Lin et al., 2014
miRNA-29c	Cultured mouse podocyte	KD	Apoptosis ↓, Caspase 3 ↓, Fibronectin ↓, IL-6 ↓, TNF-α↓	Spry1 TTP	Long et al., 2011; Guo et al., 2017
miRNA-30s	Cultured mouse podocyte	OE	Apoptosis ↓, Mtdh ↓, Bax ↓, Caspase 3 ↓	MTDH	Liu et al., 2016
miRNA-34a	Cultured mouse podocyte	OE	Apoptosis ↓, Caspase 3 ↓, Bcl-2 ↑, Bax ↓, p-p53 ↓, β-Arrestin-1 ↓, β-arrestin-2 ↓	Notch 1, Jagged 1, NICD, Hes 1, Hey 1	Zhang et al., 2016
miRNA-34c	Cultured mouse podocyte	OE	Apoptosis ↓, Notch 1 ↓, Hes1 ↓, Hey1 ↓, NICD ↓, Bcl-2 ↑, Bax ↓, Cleaved caspase-3 ↓, p-p53 ↓	Notch1, Jaggged1	Liu et al., 2015
miRNA-93	Cultured mouse podocyte	OE	CTGF ↓, Fibronectin ↓, Serpone1 ↓, Rock ↓, WT-1 ↓, α3 collagen IV ↓, Fibronectin ↓	Msk2 VEGF-A	Long et al., 2010; Badal et al., 2016
miRNA-130a-3p	Cultured mouse podocyte	OE	TNF-α↓, ROS ↓, SOD ↑, MDA ↓	TNF-α	Jiang et al., 2018
miRNA-134-5p	Cultured human podocyte	KD	Apoptosis ↓, Nephrin ↓, Cleaved caspase 3 ↓	Bcl-2	Qian et al., 2018
miRNA-145-5p	Cultured mouse podocyte	OE	Apoptosis↓, Caspase 3 ↓, Bcl-2 ↑, Bax ↓	Notch1	Wei et al., 2020
miRNA-182-5p	Cultured mouse podocyte	KD	Apoptosis↓, Cell survival rate ↑	CD2AP	Ming et al., 2019
miRNA-193a	Cultured human podocyte	KD	WT-1 ↑, PAX2 ↓	APOL1	Mishra et al., 2018
miRNA-195	Cultured mouse podocyte	KD	Bcl 2 ↑, Caspase 3 ↓, Caspase 8 ↓, WT-1 ↑, Synaptopodin ↑, Apoptosis ↓	Bcl-2	Chen et al., 2011
miRNA-217	Cultured mouse podocyte	KD	Nephrin ↑, Apoptosis ↓, Glucose uptake ↑, ROS Production ↓, Synaptopodin ↑, Podocine ↑, LC3II/LC3I ↑, Beclin-1 ↑, p62	PTEN	Sun et al., 2017
miRNA-218	Cultured mouse podocyte	OE	Nephrin ↑, Apoptosis ↓, p-p38 ↓	HO-1	Yang et al., 2016
miRNA-301a-3p	Cultured mouse podocyte	OE	TNF-α↓, ROS ↓, SOD ↑, MDA ↓	TNF-α	Jiang et al., 2018
miRNA-423-5p	Cultured mouse podocyte	OE	Proliferation ↑, Apoptosis ↓, NADPH oxidase activity ↑, ROS production ↓, IL-6 ↓, IL-1β↓, TNF-α↓, MCP-1 ↓ Cleaved caspase-3 ↓, Cleaved caspase-9 ↓, Bax ↓, Bcl-2 ↑, Bax ↓, F-actin ↑	NOX4	Xu et al., 2018
miRNA-503	Cultured mouse podocyte	KD	Migration ↓ Apoptosis ↓	E2F3	Zha et al., 2019
miRNA-770-5p	Cultured mouse podocyte	KD	Proliferation ↑ Apoptosis ↓	TRIAP1	Zhang et al., 2019
miR-874	Cultured mouse podocyte	OE	Cell survival rate ↑, Proliferation ↑, Apoptosis ↓, IL-6 ↓, IL-1β↓, TNF-α↓	TLR4	Yao et al., 2018

**TABLE 3b:** Effects of changes of miRNA expression levels on podocyte injury in diabetic nephropathy *in vivo*.

miRNA	Model	Treatment	Method on miRNA overexpression or knockdown	Biological effects	Target mRNA	References
miRNA-16-5p	STZ-induced diabetic nephropathy rats	OE	Human urine-derived stem cells exosomes containing overexpressed miR-16-5p were injected via tail vein	Podocyte number ↑, foot process width ↓, podocyte fusion rate ↓, mesangial area ↓, apoptosis ↓, TNF-α↓, TGF-β↓, MCP-1 ↓	VEGFA. Vascular endothelial growth factor A.	Duan et al., 2019
miRNA-21	STZ-induced diabetic nephropathy rats. STZ-induced diabetic nephropathy mice.	KD	anti-miR-21 were injected via tail vein.	Serum: IL-1β↓, TNF-α↓, Blood: glucose ↓ Creatinine ↓, Blood urine nitrogen ↓, Kidney: Bax ↓, Bcl-2 ↑, Apoptosis ↓, Mesangial expansion ↓, Mesangial expansion ↓, Interstitial fibrosis ↓, Collagen Ia2 ↓, Collagen III, CTGF, α-SMA) ↓, Infiltrating macrophages ↓, MCP-1 ↓ Albuminuria ↓, Podocyte loss ↓	TIMP3 Cdc25a Cdk6	Kolling et al., 2017; Chen et al., 2018
miRNA-23b	db/db mice	OE	miR-23b agomir were injected via tail vein.	Body weight ↓, Abdominal fat weight ↓, Insulin resistance index ↓ Insulin sensitivity ↑, Kidney: Microalbuminuria ↓, Albumin-to-creatinine ratio ↓, p53 ↓, p38MAPK ↓, GBM thickening ↓, Podocyte foot processes ↑	G3BP2	Zhao et al., 2016
miRNA-25	db/db mice	OE	miR-25 agomir were injected via tail vein	Proteinuria ↓ Kidney: Glomerular fibrosis ↓, α-SMA ↓, Foot process fusion of podocytes ↓, GBM thickening ↓, BP ↓, KRI ↓, angiotensin 1 area ↓, Renin area ↓, wVF ↓ serum: Renin ↓, Angiotensin I ↓, Angiotensin II ↓, Aldosterone ↓	CDC42	Liu et al., 2017
miRNA-27a	STZ-induced diabetic nephropathy rats	KD	miR-27a inhibitor were injected intraperitoneally.	Renal function ↑, Proteinuria ↓ Kidney: Podocyte number ↑, Effaced podocyte foot processes ↓, PPAR-γ↓, β-catenin ↓, snail1 ↓, α-SMA ↓, Synaptopodin ↑, WT-1 ↑	PPAR-γ	Zhou et al., 2017
miRNA-29a	STZ-induced diabetic nephropathy in miRNA-29a transgenic mice	OE	miRNA-29a transgenic mice	Serum: Diabetes-induced hyperfiltration ↓, Creatinine clearance ↑ Kidney: Kidney weight ↓, Urinary protein levels ↓, Glomerular hypertrophy ↓, Desmin ↓, Apotosis ↓, Nephrin ↑, WT-1 ↑, TGF-β1 ↓, Fibronectin ↓, IL-1β↓	HDAC4	Lin et al., 2014
miRNA-29c	db/db mice	KD	Chemically modified antisense RNA oligonucleotides were injected intraperitoneally.	Kidney: Albuminuria ↓, Apoptosis ↓, Mesangial matrix accumulation ↓, Fibronectin ↓	Spry1	Long et al., 2011
miRNA-93	miR-93 transgenic db/db mice	OE	miRNA-93 transgenic mice	Kidney: Albuminuria ↓, Mesangial matrix expansion ↓, Desmin ↓, Podocyte loss ↓, Synaptopodin ↑, Nephrin ↑, Foot process effacement ↑, Slit diaphragm morphology ↑, Glomerular basement membrane thickness ↓, Foot process effacement ↓, Foot process density ↑	Msk2	Badal et al., 2016
miRNA-146a	STZ-induced diabetic nephropathy in miR-146a deficient mice.	KD	miR-146a-deficient mice.	Kidney: Mesangial sclerosis ↓, Foot process effacement ↓, EGFR ↓	ErbB4 Notch-1	Lee et al., 2017
miRNA-503	STZ-induced diabetic nephropathy rats.	KD	miR-503 inhibitor was injected intravenously.	Proteinuria ↓, Serum creatinine ↓, Blood urea nitrogen ↓	E2F3	Zha et al., 2019
miR-874	STZ-induced diabetic nephropathy mice.	OE	Adenovirus carrying miR-874 were injected intravenously	Body weight ↑, Proteinuria ↓ Serum: Creatinine ↓, Blood urea nitrogen ↓ Kidney: IL-6 ↓, IL-1β↓, TNF-α↓	TLR4	Yao et al., 2018

## miRNA-16-5p

High glucose stimulation could inhibit miR-16-5p and promote VEGFA in human podocytes. The OE of miR-16-5p using human urine−derived stem cells (hUSCs) could improve the viability and apoptosis rate of podocytes after high-glucose treatment *in vitro*. Furthermore, the OE of miRNA-16-5p using hUSC exosomes containing overexpressed miR-16-5p was shown to attenuate podocyte injury in diabetic nephropathy rats. It has been suggested that the protective effect of miRNA-16-5p against podocyte injury occurs through the inhibition of VEGF A, which has been reported to be an important mediator of diabetic nephropathy and is involved in the endogenous angiogenesis of endothelial cells (Duan et al., 2019).

## miRNA-20b

The KD of miRNA-20b using a specific inhibitor of it carried by liposomal nanoparticles was shown to inhibit podocyte apoptosis by increasing the expression of sirtuin 7, a target mRNA of miRNA-20b, in cultured mouse podocyte cells stimulated with a high glucose concentration (Wang et al., 2017).

## miRNA-21

The OE of miRNA-21 using miRNA-21 mimic was shown to decrease the expression levels of nephrin and increase the expression levels of α-SMA in cultured mouse podocyte cells with activation of the β-catenin pathway, and the TGF-β_1_/Smads pathway (Wang et al., 2018). Meanwhile, the KD of miRNA-21 expression dramatically reduced podocyte migration in cultured podocyte cells by regulating phosphatase and tensin homolog (Pten) (Kolling et al., 2017). Another study reported that the KD of miRNA-21 using a specific inhibitor of it decreased the expression levels of pro-inflammatory cytokines such as IL-1β and TNF-α, and alleviated kidney damage in STZ-induced diabetic nephropathy rats (Chen et al., 2018). These lines of evidence suggest that miRNA-21 promotes podocyte injury in diabetic nephropathy.

## miRNA-23b

The OE of miRNA-23b in kidney by the weekly intravenous injection of miR-23b mimic was shown to have protective effects against diabetic nephropathy by improving podocyte injury, including flattening of the foot processes resulting in decreased microalbuminuria in a diabetic nephropathy mouse model (Zhao et al., 2016). In contrast, the inhibition of miRNA-23b expression in kidney by intravenous injection with miR-23b inhibitor was shown to increase kidney fibrosis, podocyte collapse, and proteinuria in wild-type mice (Zhao et al., 2016). The mechanism behind the protective effects of miRNA-23b in diabetic nephropathy has been suggested to occur via inhibition of the production of extracellular matrix proteins in diabetic nephropathy via the activation of p38 MAPK. In addition, Ras GTPase-activating protein SH3 domain-binding protein 2, which is considered a target mRNA of miRNA-23b, forms an miR-23b/G3BP2 feedback circuit involving p38 MAPK and p53 (Zhao et al., 2016). These lines of evidence suggest that miRNA-23b has protective effects against podocyte injury in diabetic nephropathy.

## miRNA-25

The OE of miRNA-25 by intravenous injection of an miR-25 mimic via the tail vein was shown to alleviate podocyte injury in a diabetic mouse model *in vivo* (Liu et al., 2017). In contrast, KD of the expression of miRNA-25 by the intravenous injection of an miR-25 inhibitor via the tail vein was shown to cause podocyte injury in wild-type mice (Liu et al., 2017). The mechanism behind the protective effects of miRNA-25 in diabetic nephropathy has been suggested to occur via the inhibition of Ras-related genes including CDC 42, which is a downstream effector of Ras and Rap1a/b primarily acting to modulate mitochondrion-derived oxidative stress (Liu et al., 2017). These lines of evidence suggest that miRNA-25 would have protective effects in podocyte injury in diabetic nephropathy.

## miRNA-27a

The KD of miRNA-27a was shown to ameliorate podocyte injury including apoptosis and decrease the levels of migration and invasion of cultured podocyte cells stimulated with a high glucose concentration *in vitro* (Zhou et al., 2017). Furthermore, KD of miRNA-27a expression via the intraperitoneal administration of miRNA-27a inhibitor was shown to mitigate podocyte injury, such as a decrease in their number, disruption of their architectural integrity, and effacement of foot processes, in a diabetic nephropathy rat model (Zhou et al., 2017). In contrast, the OE of miRNA-27a by the intraperitoneal administration of an miR-27a mimic was shown to exaggerate podocyte injury in a diabetic nephropathy rat model (Zhou et al., 2017). The mechanism behind the protective effects of miRNA-27a against podocyte injury in diabetic nephropathy has been suggested to involve increases in proliferator-activated receptor-γ (PPAR-γ) and activated β-catenin (Zhou et al., 2017). PPAR-γ phosphorylation induces β-catenin activation and triggers a series of β-catenin-dependent reprogramming events, including enhanced epithelial–mesenchymal transition, loss of podocyte-specific markers, and increased apoptosis (Zhou et al., 2017).

## miRNA-29a

The KD of miRNA-29a using an antisense oligo of it was shown to promote the apoptosis and nephrin loss of podocytes in mice without diabetes mellitus (Lin et al., 2014). In contrast, the OE of miRNA-29a was shown to attenuate apoptosis in cultured podocyte cells stimulated with a high glucose concentration (Lin et al., 2014). The mechanism behind the protective effects of miRNA-29a against podocyte injury has been suggested to occur through the attenuation of histone deacetylase4-dependent nephrin deacetylation and ubiquitination, which accelerates podocyte apoptosis (Lin et al., 2014).

## miRNA-29c

The KD of miRNA-29c using a specific inhibitor of it carried by a viral vector was shown to inhibit apoptosis and reduce inflammatory cytokines, mediated by OE of its target mRNAs [Spry1 and TTP] in cultured mouse podocyte cells (Long et al., 2011; Guo et al., 2017). Spry1 is known to inhibit the Ras/MEK/ERK pathway (Kim and Bar-Sagi, 2004). It has also been identified as a negative regulator of Rho A and its downstream effector Rho kinase, which plays a key role in diabetic nephropathy through non-canonical Wnt signaling (Miyoshi et al., 2004). In addition, TTP is a regulator of the stability of TNF-α mRNA (Deleault et al., 2008). Furthermore, KD of miRNA-29c by a chemically modified antisense oligonucleotide was shown to reduce albuminuria and mesangial matrix accumulation in diabetic nephropathy mice *in vivo* (Long et al., 2011). These lines of evidence suggest that miRNA-29c would promote podocyte injury in diabetic nephropathy.

## miRNA-30s

The OE of miRNA-30s using miRNA-30s mimic carried by liposomal nanoparticles was shown to attenuate the apoptosis of murine podocyte cells stimulated with a high glucose concentration *in vitro* (Liu et al., 2016). Inhibiting MTDH, a target of miRNA-30s that regulates apoptosis through the p38 MAPK pathway, which has an anti-apoptotic effect in TNF-α-induced apoptosis, was considered to be a mechanism behind the protective effects of miRNA-30c against injury to podocyte cells stimulated with a high glucose concentration *in vitro* (Liu et al., 2016).

## miRNA-34a

The OE of miRNA-34a using an miRNA-34a mimic carried by liposomal nanoparticles was shown to inhibit apoptosis in cultured podocyte cells stimulated with a high glucose concentration *in vitro* by inhibiting the Notch signaling pathway (Zhang et al., 2016).

## miRNA-34c

The OE of miRNA-34c by an miRNA-34c mimic carried by liposomal nanoparticles was shown to inhibit apoptosis in cultured podocyte cells stimulated with a high glucose concentration *in vitro* (Liu et al., 2015). Inhibiting the Notch signaling pathway by targeting Notch1 and Jaggged1 by miRNA-34c was assumed to be a mechanism behind the inhibition of apoptosis by miRNA-34a in podocyte cells stimulated with a high glucose concentration *in vitro* (Liu et al., 2015).

## miRNA-93

The OE of miRNA-93 was shown to attenuate podocyte injury in diabetic nephropathy mice *in vivo* (Badal et al., 2016). The protective mechanism of miRNA-93 against podocyte injury has been suggested to occur through inhibition of the mitogen- and stress-activated protein kinase 1 (Msk2), which is a kinase for Histone H3 Ser10 phosphorylation (H3S10P) and can lead to widespread changes in chromatin organization and gene transcription (Badal et al., 2016). Another study reported that the OE of miRNA-93 by an miRNA-93 mimic in cultured podocyte cells decreased the expression levels of collagen type IV alpha 3 and fibronectin, which are downstream targets of vascular epithelial growth factor, a target mRNA of miRNA-93, *in vitro* (Long et al., 2010). These lines of evidence suggest that miRNA-93 has protective effects against diabetic nephropathy.

## miRNA-130a-3p/301a-3p

The OE of miRNA-130a-3p/301a-3p using an miRNA-130a-3p/301a-3p mimic carried by liposomal nanoparticles was shown to attenuate apoptosis by inhibiting TNF-α in cultured mouse podocyte cells stimulated with a high glucose concentration *in vitro* (Jiang et al., 2018).

## miRNA-134-5p

The OE of miRNA-134-5p using an miRNA-134-5p mimic was shown to increase apoptosis in cultured human podocyte cells by inhibiting the expression of BCL-2, an apoptosis-related gene. The inhibition of miRNA-134-5p was also shown to have the opposite effect (Qian et al., 2018).

## miRNA-145-5p

The OE of miRNA-145-5p using miRNA-145-5p mimic carried by liposomal nanoparticles inhibited high-glucose-induced apoptosis *in vitro* by suppressing the high-glucose-induced activation of Notch1, which is a vital factor in the Notch signaling pathway and plays an important role in podocyte apoptosis (Wei et al., 2020).

## miRNA-146a

miRNA-146a-deficient mice were shown to exhibit more accelerated development of albuminuria, glomerular sclerosis, and podocyte injury including foot process effacement of podocyte cells upon the induction of diabetes mellitus by STZ administration (Lee et al., 2017). Erlotinib, an inhibitor of the epidermal growth factor family inhibitor, was shown to significantly decrease podocyte injury, including protection against foot process effacement, and suppress the reduction in podocyte numbers, in miRNA-146a-deficient animals upon the induction of diabetes mellitus by STZ administration (Lee et al., 2017).

## miRNA-182-5p

The apoptosis of cells was suppressed by miRNA-182-5p inhibitor carried by liposomal nanoparticles and promoted by miR-182-5p mimics. In addition, the survival rate of cells was the highest after transfection with miR-182-5p inhibitor *in vitro*.

The protective effect of miRNA-182-5p on podocyte injury has been suggested to occur via a negative regulatory effect of miR-182-5p on CD2AP, which is associated with the development of diabetic nephropathy (Ming et al., 2019).

## miRNA-193a

The KD of miRNA-193a by an miRNA-193a inhibitor carried by liposomal nanoparticles was shown to maintain the phenotype of podocyte cells by inhibiting dedifferentiation in cultured podocyte cells stimulated with a high glucose concentration *in vitro* (Mishra et al., 2018). Silencing of APOL1, via the inhibition of miRNA-193a, is considered to be a mechanism of preserving the phenotype of podocyte cells (Mishra et al., 2018).

## miRNA-195

The OE of miRNA-195 by an miRNA-195 mimic was shown to increase apoptosis via enhancing caspase cascades in cultured immortalized mouse podocyte cells *in vitro* (Chen et al., 2011). In contrast, miRNA-195 inhibitor was shown to provide significant protective effects against the apoptosis of podocyte cells (Chen et al., 2011).

## miRNA-217

The KD of miRNA-217 expression by a specific inhibitor of it carried by liposomal nanoparticles was shown to attenuate decreased viability and cell apoptosis in cultured podocyte cells stimulated with a high glucose concentration *in vitro* (Sun et al., 2017). These protective effects of miRNA-217 were shown to occur through the autophagy pathway via targeting phosphatase and tensin homolog deleted from chromosome 10, which can negatively regulate PI3K/AKT/mTOR signaling, subsequently enhancing autophagy and being a target mRNA of miRNA-217 (Sun et al., 2017).

## miRNA-218

The OE of miRNA-218 using a miRNA-218 mimic carried by liposomal nanoparticles was shown to increase apoptosis in cultured immortalized mouse podocyte cells stimulated with a high glucose concentration. The inhibition of miRNA-218 was also shown to have the opposite effects *in vitro* (Yang et al., 2016). These effects of miRNA-218 on podocyte cells were shown to be caused by the inhibition of heme oxygenase (HO), which is involved in antioxidant defense and is a key cytoprotective enzyme, and the activation of p38-mitogen-activated protein kinase (MAPK), a pro-apoptotic molecule and target mRNA of miRNA-218 (Yang et al., 2016).

## miRNA-423-5p

The OE of miRNA-423-5p using an miRNA-423-5p mimic carried by liposomal nanoparticles was shown to inhibit apoptosis by reducing reactive ROS production, migratory activity, and cytoskeletal damage in cultured immortalized mouse podocyte cells stimulated with a high glucose concentration *in vitro* (Xu et al., 2018). These protective effects of miRNA-423-5p against podocyte injury were shown to be caused by the inhibition of NADPH reduced oxidase 4 (Nox4), which is a target mRNA of miRNA-423-5p (Xu et al., 2018). Nox4 is characterized by the constitutive release of hydrogen peroxide, which has been identified as the major enzymatic source of ROSs in diverse renal cells (Sedeek et al., 2013).

## miRNA-503

The podocyte apoptosis stimulated with a high glucose concentration was strongly repressed by the downregulation of miRNA-503 carried by liposomal nanoparticles *in vitro*. Furthermore, intravenous injection of miR-503 inhibitor into diabetic rats resulted in decreases of proteinuria, serum creatinine, and BUN levels. These effects of miRNA-503 were shown to be caused by the inhibition of E2F3, which modulates various cellular processes, such as apoptosis, differentiation, and development, with E2F1, 2, and 3 as the transcriptional activators (Zha et al., 2019).

## miRNA-770-5p

High-glucose-induced cell apoptosis and decreased cell proliferation ability were significantly inhibited by miR-770-5p inhibitor using liposomal nanoparticles *in vitro*. The suppression of TRIAP1 by miR-770 exerts this protective effect on podocytes (Zhang et al., 2019).

## miRNA-874

The OE of miRNA-874 was shown to increase the survival and proliferative ability of cultured mouse podocyte cells stimulated with a high glucose concentration by inhibiting the expression level of toll-like receptor 4 (TLR4), a target mRNA of miRNA-874 *in vitro* (Yao et al., 2018). TLR4 is reported to be upregulated in diabetic patients, and it can increase the expression of interleukin-6 (IL-6), tumor necrosis factor-α (TNF-α), and IL-1β. Additionally, the OE of miRNA-874 in kidney by injecting adenovirus carrying an miR-874 mimic was shown to significantly decrease the infiltration of inflammatory cells and the expression levels of inflammatory cytokines such as IL-6, IL-1β, and TNF-α with inhibition of the expression of TLR4 in kidney, resulting in decreased albuminuria and increased renal function in a rat diabetic nephropathy model *in vivo* (Yao et al., 2018).

## Discussion

This review focuses on miRNAs in podocyte injury in diabetic nephropathy, with regard to their potential as biomarkers and miRNA modulation as a therapeutic option. miRNAs have been shown to play pivotal roles in the development of podocyte injury in diabetic nephropathy, indicating that they may represent potential biomarkers and therapeutic options. Further clinical studies are needed to investigate their usefulness in detail.

## Author Contributions

HI drafted the manuscript. SK, KY, and AA participated in the discussion and revision of this manuscript. KH, SO, and KI contributed critical analysis. YM conceived the whole program, and extensively revised the manuscript. All authors contributed to the article and approved the submitted version.

## Conflict of Interest

The authors declare that the research was conducted in the absence of any commercial or financial relationships that could be construed as a potential conflict of interest.

## Abbreviations

APOL1: apolipoprotein L1
Bcl-2: B-cell lymphoma 2
BP: blood pressure
BUN: blood urea nitrogen
CD2AP: CD2-associated protein
CTGF: connective tissue growth factor
E2F3: E2F transcription factor 3
ErbB4: Erb-b2 receptor tyrosine kinase 4
G3BP2: G3BP stress granule assembly factor 2
GBM: glomerular basement membrane
HDAC: histone deacetylase
HO-1: heme oxygenase-1
IL: interleukin
KD: knockdown
KRI: kidney artery resistance
MCP-1: monocyte chemotactic protein
MDA: malondialdehyde
Msk2: mitogen and stress-activated kinase
MTDH: metadherin
NADPH: nicotinamide adenine dinucleotide phosphate
NICD: notch intracellular domain
NOX: nicotinamide adenine dinucleotide phosphate oxidase
OE: overexpression
PPAR-γ: peroxisome proliferator-activated receptor- γ
PTEN: phosphatase and tensin homolog deleted on chromosome 10
Rock, rho-associated: coiled-coil-containing protein kinase 1
ROS: reactive oxygen species
SIRT7: sirtuin7
SMA: smooth muscle actin
SOD: superoxide dismutase
Spry1: sprouty homolog 1
STZ: streptozotocin
TGF: transforming growth factor
TIMP: tissue inhibitor of metalloproteases
TLR: toll-like receptor
TNF: tumor necrosis factor
TRIAP1: TP53-regulated inhibitor
TTP: tristetraprolin
TUNEL: TdT-mediated dUTP nick end labeling
UACR: urine albumin to creatinine ratio
UAER: urine albumin excretion rate
VEGF: vascular endothelial growth factor
WT-1: Wilm’s tumor protein 1
wVF: Von Willebrand factor.
